# A Case of Periodical Strabismus

**Published:** 1861-10

**Authors:** F. N. Smith

**Affiliations:** LaHarpe, Ill.


### ARTICLE V.

### A CASE OF PERIODICAL STRABISMUS.

By F. N. SMITH, M. D., LaHarpe, Ill.

A son of Mr. Brockway, of this place, aged four years, was attacked a year ago last March, with dysentery. He only suffered from it about a week. At the time convalescence from this disease commenced, he became affected with convergent strabismus. This caused so much anxiety on the part of his parents, that they consulted their physician with reference to it. He prescribed alteratives and tonics. Under this treatment, the strabismus gradually subsided, and I believe he was considered perfectly free from it, for three or four weeks, when it returned, but periodically, occurring on every alternate day. He was now treated with anti-periodics. Quinine was first used,—this failed. Arsenic and strychnine were successively used, without any benefit ensuing, though the latter remedy was given till its constitutional effect was produced. All treatment was then suspended. He has taken nothing for over a year. Up to three months ago, the strabismus continued much the same, strictly periodical. The child's health has continued good, with very slight exceptions, from the time of attack of dysentery alluded to in the outset. His mother says he appears more fretful on the day his eyes are affected, than on the preceding day. Of late, the strabismus has shown a tendency to occur every day. In fact, occasionally, for several days in succession, his eyes will be affected every day, with scarcely any difference in their appearance; then for some weeks, perhaps, the periodical character of the affection will be prominent. His appetite is excellent, and he sleeps well. His head is of good size, and his body well developed.

I submit the questions: What is the cause of the periodicity in this case? Is it connected with malaria? If so, why does it not yield to the most powerful anti-periodics?

The especial object of this article is to elicit comment on this singular case, in regard to its cause and treatment.

I have two melanotypes of the little boy; one taken on a day on which his eyes were affected, and the other on the following day.

